# Fiber optics-based surface enhanced Raman Spectroscopy sensors for rapid multiplex detection of foodborne pathogens in raw poultry

**DOI:** 10.1038/s41378-024-00834-x

**Published:** 2024-12-23

**Authors:** Mai Abuhelwa, Arshdeep Singh, Jiayu Liu, Mohammed Almalaysha, Anna V. Carlson, Kate E. Trout, Amit Morey, E. Kinzel, Lakshmikantha H. Channaiah, Mahmoud Almasri

**Affiliations:** 1 Department of Electrical Engineering and Computer Science, University of Missouri, Columbia, MO 65211 USA; 2 Division of Food, Nutrition & Exercise Sciences, University of Missouri, Columbia, MO 65211 USA; 3 Cargill, Inc, Wichita, KS 67202 USA; 4 College of Health Sciences, University of Missouri, Columbia, MO 65211 USA; 5 Department of Poultry Science, Auburn University, Auburn, AL 36849 USA; 6 Mechanical and Aerospace Engineering, University of Notre Dame, Notre Dame, IN 46556 USA

**Keywords:** Optical sensors, Biosensors

## Abstract

A new high-sensitivity, low-cost, Surface Enhanced Raman Spectroscopy (SERS) sensor allows for the rapid multiplex detection of foodborne pathogens in raw poultry. Self-assembled microspheres are used to pattern a hexagonal close-packed array of nanoantennas onto a side-polished multimode fiber core. Each microsphere focuses UV radiation to a photonic nanojet within a layer of photoresist on the fiber which allows the nanoantenna geometry to be controlled. Optimizing the geometry for the excitation layer generates electric field concentrations− referred to as a hotspot− within the analyte, thereby maximizing the Raman signal and improving the signal-to-noise ratio. The side polished configuration with a larger surface area has significantly better performance than the SERS sensor on the fiber tip. The use of additive manufacturing for the fiber polishing jigs as well as the sample testing compartment simplifies the sensor development and testing. Experimental results demonstrate a sensitivity range of 0.4–0.5 cells/ml achieved using raw chicken rinsates spiked with *Salmonella* typhimurium. Additionally, the sensor demonstrated its capability for multiplex and specific detection of *Salmonella* and *E. coli O157:H7* with an optimal detection time of 10 min. The new sensor addresses a major global foodborne pathogen that poses significant public health concerns and can be readily adapted for the detection of other bacterial and viral pathogens such as *E. coli O157:H7*, *Campylobacter*, *Listeria*, and avian influenza and in other food products, e.g., dairy, beef, and produce, as well as clinical applications.

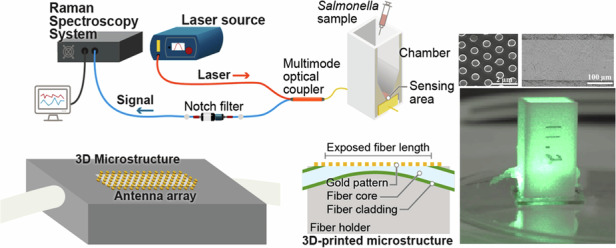

## Introduction

*Salmonella* stands as a leading cause of foodborne illness in both the U.S. and globally^1^. Annually, it results in 1.35 million infections, 26,500 hospitalizations, and 420 deaths in the United States alone^2^. Despite national goals for improvement, this incidence has remained unchanged for three decades in the U.S^3^. The primary reservoirs of *Salmonella* in animals are their intestines, leading to most *Salmonella* illnesses being attributed to cross-contamination during slaughter, processing undercooking of raw meat poultry products, and due to environmental contamination^4^. Poultry meat, encompassing chicken and turkey products, has been identified by the CDC as the predominant source of *Salmonella* infections^5^, accounting to approximately 23.4% of salmonellosis cases^6^. The Center for Disease Control and Prevention (CDC 2023) estimates that one out of every 25 chicken packages sold in the grocery store is contaminated with *Salmonella*. The economic impact of *Salmonella* alone on the U.S. accounts for $4.1 billion annually for medical costs, loss of productivity, and deaths^1^.

Current *Salmonella*-detection methods rely heavily on Polymerase Chain Reaction (PCR)^7^, which requires at least 24 h (including enrichment, sample preparation, and nucleic acid extraction) to detect *Salmonella*, and another 5–7 days for confirmation of positive status of the samples using traditional microbiological culture methods^8,9^. Although regarded as gold standards for the detection of pathogens in clinical and food products, traditional and PCR-based methods are time-consuming and expensive^8^. Additionally, immunological methods such as ELISA has a rapid detection step but is done after an enrichment culture^10^, However, the lack of timely results from current pathogen detection methods hinders poultry manufacturers from promptly implementing *Salmonella* intervention steps to enhance food safety. As such, various rapid, foodborne pathogen detection methods have been investigated over the last two decades such as electrochemical^11^, impedance^12,13^, optical^14^, lateral flow assay^15^, potentiometric^16^, and quartz crystal microbalance^17^. The impedance sensing technique was combined with focusing and trapping regions to concentrate low quantities of pathogens (e.g., *Salmonella*) to a detectable threshold, capture and detect *Salmonella*, achieving LOD of 7–10 CFU/ml for *Salmonella* serotypes in raw chicken and ready to eat turkey samples^18,19^, graphene to provide high carrier mobility to detect *E. coli* O157:H7 between 10−100 cells/ml^20^. A potentiometric biosensor based on single-walled carbon nanotubes with immobilized aptamers detected *E. coli* O157:H7 in apple juice with a LOD 26 CFU/ml^21^. Paper-based techniques provided a LOD 30–300 CFU/mL in food matrices with eye readout^22^. Optical biosensors such as surface plasmon resonance with Fe_3_O_4_ magnetic nanoparticles separation achieved LOD of 14 CFU/mL^23^, and many other techniques^24–26^. However, these methods often require significant instrumental inputs, sample preparation time, and high cost.

The SERS sensors were fabricated on flat substrate or fiber tip by creating various roughened nanostructure surfaces using chemical synthesis, photolithography, nanoimprint lithography, or nanosphere lithography. Chemical synthesis techniques involving metal nanoparticles (NPs), such as gold (Au^27^) NPs of varying shapes, sizes, and aggregates^28–33^ (e.g., stars^34^), have been explored to improve detection sensitivity. While NPs have enhanced the Raman signal by several orders of magnitude, their random distribution across the substrate can lead to uncontrollable hotspots, limiting the potential for maximizing the Raman signal^35^. Photolithography techniques, such as electron beam (E-Beam) lithography, can produce uniform patterns^29^, e.g., nano-disk^36^, but they are expensive and not practical for large-scale sensor production. An alternative low-cost technique, polystyrene nanosphere lithography^37^, uses nanospheres as templates to create nano-islands and nano-cavities^38^, but the size control of patterns remains challenging. SERS sensing has been used for many applications, including the detection of Zika virus, dengue NS1 biomarkers^39^, and SARS-CoV-2 spike protein at a concentration of 6.07 fg/mL in untreated saliva^40^. While SERS measurements have yielded promising results, they still require bulky optical systems, which limit their practicality.

The excitation light in the optical fibers-based SERS sensors is delivered through the fiber and the scattered Raman signal is collected through the same fiber using a bidirectional coupler. To enhance the Raman signal, optical fibers of various shapes and types, such as fiber tip^41^, hollow photonic crystal fiber (PCF)^42^, D-shaped fiber^43^, and tapered fiber^44^ were used. For example, hollow fiber increases the sample volume that interacts with light and NPs such as Ag NPs^45^. However, applying the sample into the air holes in the PCF is challenging. The D-shaped fiber still necessitates the use of a Raman microscope. While sensors on flat substrates have shown excellent Raman signal enhancement, fiberoptic SERS sensors are preferred for their portability, compact size, high stability, system integration, fiber flexibility, robustness, remote sensing, capabilities^46^, and distributed sensor network. In this paper, the sensor was tested for detection of *Salmonella* and *E. coli* O157: H7 simultaneously, making it a highly effective diagnostic tool for poultry processing plant applications.

Raman spectroscopy, particularly SERS, is a powerful analytical tool that can identify molecules based on their composition and structure. SERS is highly sensitive, rapid, label-free, and can probe solutions in the visible and near-infrared regions due to inelastic scattering of light when interacting with molecules. It offers a plethora of label free and non-invasive diagnostic applications for molecular sensing including disease diagnosis, trace analysis, and biological, environmental pollutants, foodborne pathogen sensing, explosive and nucleic acid targets^47–52^. However, Raman scattering is generally weak since the Raman scattering is a higher order non-linear process with a lower probability than absorption spectroscopy, which limits the signal intensity of Raman spectra. To address this issue and enable detection of low concentrations of analytes, SERS surfaces were utilized^53^. These surfaces are purposefully roughened to amplify Raman scattering signals by several orders of magnitude^54^, enabling detection of low concentration of analytes^55^.

Through use-inspired research, the development of the biosensor is directly informed by real-world needs, involving stakeholders across the entire poultry supply chain and health outreach sectors, underscoring the need for a cost-effective, rapid, and easy-to-use sensor for *Salmonella* detection. This manuscript discusses the design, fabrication, and testing and validation of a fiberoptic based SERS sensors to detect *Salmonella* contamination, in 10 min, directly from chicken rinsates samples without enrichment, enabling timely data-based food safety decisions. The measured fingerprint Raman spectra contains specific peaks that represent the molecular composition of *Salmonella* or other pathogens, such as proteins, lipids, nucleic acids, and cell wall components. The sensor will be incorporated into a comprehensive sensor-enabled decision support system designed to address significant food safety challenges and enhancing public health outcomes.

## Materials and methods

### Biosensor design

**Fig. 1:**
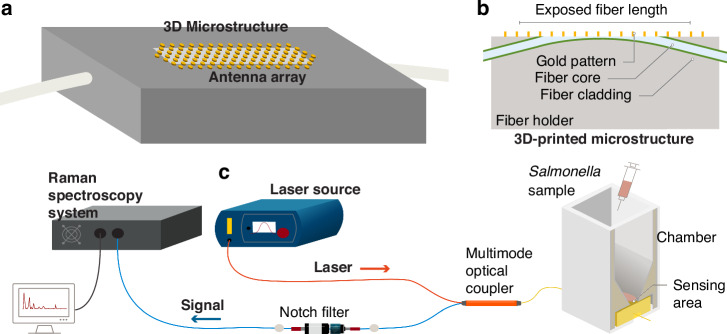
SERS sensor (**a**) 3D view, (**b**) sideview, (**c**) setup. Raman instrument is used in backscattered configuration

The Surface Enhanced Raman Spectroscopy (SERS) sensor was designed for sensitive detection and identification of *Salmonella* in poultry products, shown in Fig. 1. The sensor incorporates for the first-time metal nanoantenna arrays on a side polished multimode optical fiber core while it is integrated within a 3D printed plastic microstructure to enable accurate detection of *Salmonella*. Specific *Salmonella* detection is achieved with high sensitivity in <10 min. This is achieved by detecting the fingerprint Raman spectra of *Salmonella*, which is primarily based on the molecular composition/ constituents of the bacteria, such as proteins, lipids, nucleic acids, and cell wall components. These molecules exhibit specific vibrational modes that scatter the laser light, generating distinct Raman spectral patterns. A significant advantage of this sensor is its ability to detect *Salmonella* without the use of antibodies.

The device design incorporates several innovative features, as shown in Fig. 1a, b: (1) A multimode optical fiber (MMF, Thorlabs, FG105LVA) is inserted into a specially designed 3D printed microstructure with 15^o^ tilted cylindrical ports (not shown in the schematic). This configuration maximizes the reflected light following the interaction of the excitation laser (Laser-532-LAB-FC, Ocean Insight) with the nanoantenna arrays and the spiked chicken sample with *Salmonella*, which is collected by a Raman spectrometer (Enhanced QEP06360 RAMAN, Ocean Insight) which has a slit width of 10 µm and a grating groove density of 1200 lines/mm. (2) A groove for side polishing was included on the top surface of the microstructure to facilitate the side polishing of the optical fiber core. The groove is rectangular with areas of 125 µm × 4 mm or 125 µm × 7 mm. Alumina polishing sheets with a grain size of 0.05 µm are used to ensure a flat and smooth surface, which is crucial for patterning the nanodisk arrays. To secure the optical fiber within the ports and maintain its position inside the trench, both the groove and the fiber ports are filled with glue before inserting the fiber through the ports. (3) Highly uniform and repeatable nanodisk arrays made of thin gold film are patterned on the polished side of the fiber core. Each nanodisk array is made up of circular disk patterns arranged in a hexagonal closed-packed lattice to form antennas, where the disk diameter and periodicity allow for optimizing the resonance wavelength and SERS signal from the *Salmonella*. The nanodisk arrays are used to roughen the sensing surface, resulting in a large field intensity and the formation of SERS hotspots through laser excitation between the disk arrays. As a result, Raman signals from *Salmonella* or other pathogens in the vicinity of the hotspots are significantly enhanced.

The SERS, basically, utilizes nanoantenna to amplify the weak Raman scattering signal by several orders of magnitude, enabling the detection of *Salmonella* spectra with high intensity peaks even at low concentrations. The SERS sensors with sensing surface area of 4 mm × 105 µm, 7.0 mm × 105 µm, and disk diameters of 615 nm, 693 nm, and 770 nm at fixed periodicity of 1 µm were tested. We have chosen to arrange the nanoantenna arrays on the polished side of the optical fiber core as a novel solution aimed at increasing the sensing surface area, thereby increasing the interaction length between the laser and the virus sample. This approach has led to a larger amount of reflected and collected SERS photons, ultimately resulted in a stronger signal to noise ratio (S/N) compared to a SERS sensor located on the fiber tip, which has a very small cross-sectional area. With this design, laser light could be lost along the length of the fiber. However, the reflected light was sufficient to generate a strong spectral signal and achieve low limit of detection. Microsphere photolithography (MPL) was employed to create the nanoantenna arrays on a side-polished multimode optical fiber core, addressing the high fabrication costs that have previously impeded the implementation of plasmonic fiber sensors. The multimode fiber employed in this study features core and cladding diameters of 105 μm and 125 μm, respectively, with a fiber length of 10 cm.

### Fabrication

**Fig. 2:**
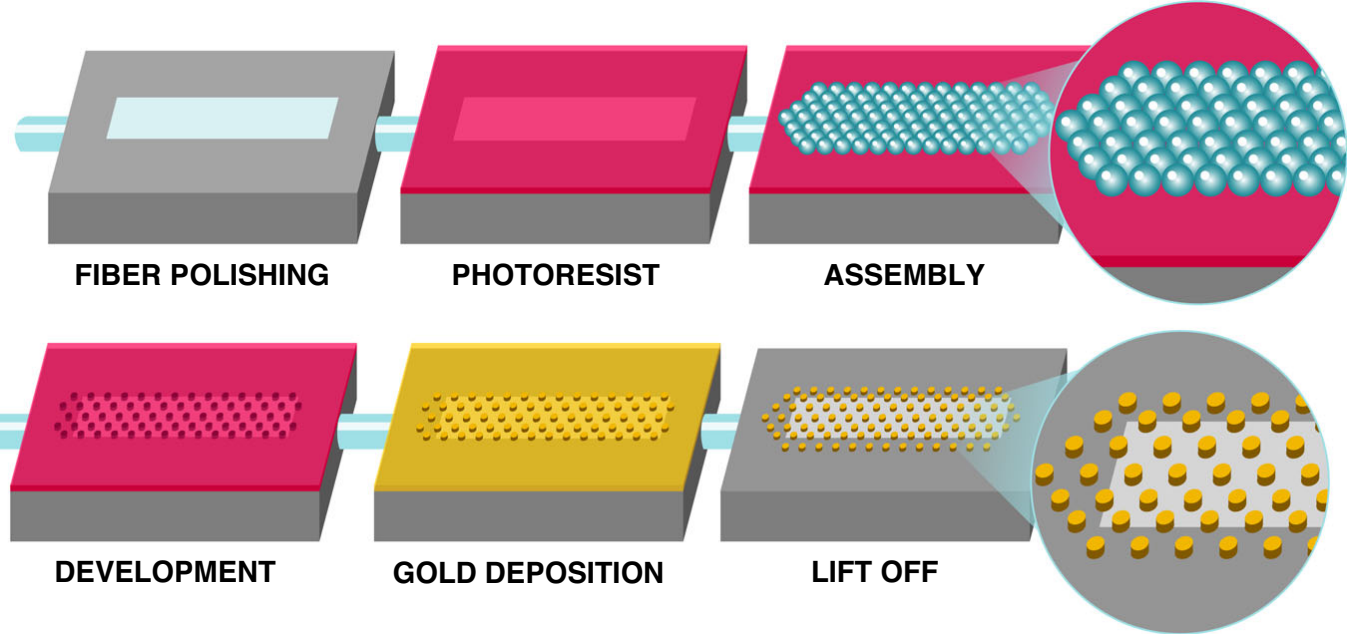
Schematics of the MPL process

The Nanoantenna arrays were fabricated on the side-polished multimode optical fiber core using MPL, a technique capable of creating highly ordered periodic structures in 2-D, as shown in Fig. 2 and as follows. First, the jacket of a multimode optical fiber was stripped off in the middle portion of the fiber with lengths of 4 mm and 7 mm, exposing the cladding for polishing purposes. A 3D-printed plastic microstructure was printed using a 3D printer (Prusa i3 Mk3 + ) with PLA Pro material (Overture), ensuring that the cylindrical ports were positioned on the sides with a 15° tilted angle and a groove on the top surface. The fiber was then inserted into the input port and emerged from the output port, aligning the exposed cladding region manually to be visible from the groove. The fiber was secured using a small amount of standard glue and baked at 110 °C. Subsequently, the fiber was polished from the groove side using 5 µm, 3 µm, 1 µm, 0.3 µm, and 0.05 µm carbide polishing paper to achieve a flat and smooth surface. The polishing depth of the fiber is not highly precise due to the manual nature of the process. The polishing process have been optimized and then applied for all fibers. While we know the fiber diameter and can measure the width of the polished area—allowing us to calculate the polished height—the goal is typically to polish down to 0.5 of the fiber core diameters (d). However, because polishing is done manually, the depth can vary slightly by 0.1 × d, typically above the target height. The polishing process begins with alumina polishing sheets, starting with a grain size of 5 µm and gradually progressing to finer sizes—3 µm, 1 µm, 0.3 µm, and finally, 0.05 µm. This stepwise approach ensures a flat and smooth surface. The polishing depth was controlled carefully by monitoring the process under an optical microscope after each step. The fiber was frequently washed and examined under the microscope. The key indicator of reaching the desired core depth is the appearance of the polishing area, combined with timing the process at each stage.

The 3D microstructure was then affixed to a glass slide, allowing the polished facet to be secured on a spin coater. The MPL process shown in Fig. 2 was employed to pattern the nanoantenna arrays on the polished surface of the fiber as follows. Initially, a thin layer of photoresist (Shipley 1805) was spin-coated onto the polished surface of the fiber to achieve a thickness of 400 nm on the fiber. After spin coating, the fiber/microstructure/glass slide assembly was removed from the chuck and immersed in a water-filled beaker. Dry silica microspheres (Sigma Aldrich) with a diameter of 1 µm and a coefficient of variance of less than 3% were dispersed in butanol to a concentration of 1 mg/ml. Ultrasonication ensured that the microsphere/butanol suspension was monodisperse. The microsphere suspension was dispensed onto the surface of a water-filled beaker. One drop (6 µL) of the microsphere suspension was dispensed onto the water surface using a syringe. Capillary forces caused the microspheres to rapidly self-assemble in a close-packed lattice on the water surface without additional interaction. The water was then slowly withdrawn by a faucet located at the bottom of the beaker, and eventually, the water surface impinged on the submerged fiber surface, transferring the microsphere lattice onto the photoresist-covered fiber surface (Fig. 2). The results showed a uniform distribution of the microspheres in a hexagonal close-packed lattice across the side of the fiber as well as a portion of the microstructure.

**Fig. 3:**
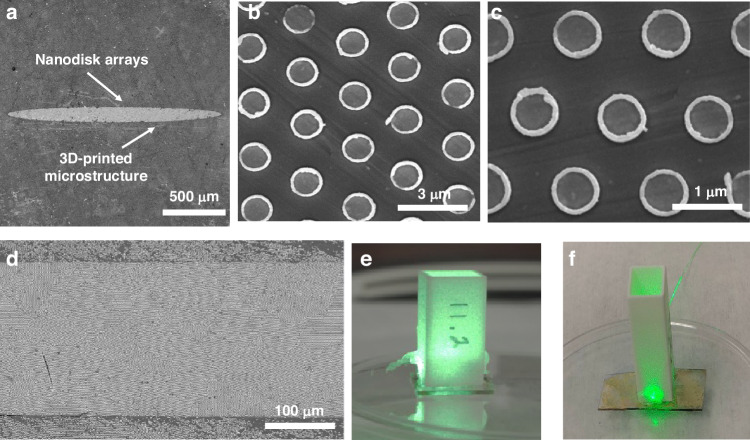
SEMs of (**a**) a 3D printed structure along with the nanodisk arrays. **b**, **c** magnified views of the nanodisks. **d** nanodisk arrays patterned across the sensing surface area. **e**, **f** Optical images of the fabricated and tested SERS sensor. The chamber, which was 3D printed, is affixed to the 3D printed microstructure with an adhesive

The photoresist was exposed to UV light at λ = 365 nm for different durations by flood illuminating with the microspheres at normal incidence using a MA6 mask-aligner. After exposure, the fiber/microstructure was developed for 20 s to remove the microspheres in the developer, washed with DI water and left to dry. This was followed by chromium (Cr) and gold (Au) layers sputter-deposition with thicknesses of 5 nm and 60 nm, respectively. The Cr/Au layer was then patterned using the lift-off process, resulting in Au nanodisk arrays with multiple diameters of 615 nm, 693 nm, and 770 nm at a fixed periodicity of 1 µm and a height of 60 nm, corresponding to exposures from 0.8 to 1.3 s. The Cr layer is used to improve the adhesion of the gold disks to the fiber surface. Additionally, the 60 nm thick Au layer was selected because, at this thickness, it effectively blocks most light transmission while still allowing patterning to form nanodisks through the lift-off process. Thicker gold films introduce challenges, as ultrasonic agitation becomes less effective in breaking the gold layer at the edges, preventing acetone from dissolving the underlying photoresist and hindering nanodisk formation. For a successful lift-off process, the photoresist layer (400 nm) must be several times thicker than the metal layer. Figure 3a–d display SEMs of the fabricated nanoantenna arrays. A 3D plastic microstructure was printed in various sizes to serve as a sample chamber. It was affixed using glue around the sensing area, as shown in the optical images in Fig. 3e, f. The SEM micrographs show a thick, rough edge forming around the disk, a result of the lift-off process, though the disk itself remains flat. In addition, in Fig. 3a, the sensing area appears elliptic, which is an artifact of the low magnification and viewing angle of the SEM. However, as depicted in Fig. 3d, the actual shape is rectangular, enclosed within a groove in the 3D-printed microstructure. This groove defines the sensor’s boundaries, with the nanodisks evenly distributed across the area. Additionally, a 3D-printed chamber was glued around the sensing area to create a sealed environment. The chamber’s inner walls were specifically designed with a tilt to direct Salmonella cells toward the sensing region, optimizing interaction between the sample and the sensing surface.

### Spiked sample preparation - procedure

Raw poultry rinsate was filtered using a 100 µm and then by a 20 µm cell strainer to remove debris (tiny meat pieces). *Salmonella* typhimurium (ATCC 14028, isolated from poultry) was used to spike the poultry rinsate. The *Salmonella* typhimurium culture was activated by transferring 0.1 ml of stock culture into 10 ml of brain heart infusion (BHI) broth and incubated at 37 °C for 24 h. Following the incubation, the grown cultures were spread-plated on five Brain-Heart Infusion (BHI) agar plates as lawns and incubated at 37 °C for 24 h. After the incubation, *Salmonella* typhimurium lawns were harvested twice using 1 ml of 0.1% peptone solution each time, and bacterial cells were dislodged using sterile plastic L-spreaders. The solution containing harvested *Salmonella* cells was then mixed with 9 ml of filtered poultry rinsate. The *Salmonella* spiked rinsates were mixed with sterile glycerol to make a 10% glycerol stock solution and serially diluted (up to 10 dilutions). After the serial dilutions, each dilution (from fifth to 10th dilution) was plated (duplicate) on XLD agar plates and incubated at 37 °C for 24 h. For the fifth to eighth dilution, 0.1 ml of the sample was plated because of the high bacterial count in the solution, whereas 1 ml of the sample was plated for the ninth and 10th dilutions because of the low bacterial count per ml. Also, for the ninth and 10th dilutions three plates were used to plate each ml of sample solution to increase the limit of detection. Immediately after the plating, each *Salmonella* dilution (up to 10 dilutions) containing 10% glycerol was frozen for further use. After incubation at 37 °C for 20–24 h, the plates were examined, counted, and labeled with *Salmonella* concentrations. Spiked samples of *Escherichia coli* O157:H7 (ATCC 43894; isolated from feces from the outbreak of hemorrhagic colitis) were prepared using a similar process.

### Experimental setup and sample testing

The SERS sensing fiber (MMF), containing the nanoantenna arrays, was fused to one end of a standard 2 × 1 multimode directional coupler (Wideband Multimode Circulator, WMC2L1F, Thorlabs, Ann Arbor, Michigan, USA). A 3D printed chamber was securely attached to the sensing fiber, enclosing the nanoantenna arrays on the fiber’s core and fraction of the cladding, as illustrated in Fig. 1c. A green laser with a wavelength of 532 nm is fed through a standard 2 × 1 multi-mode directional coupler for 10 min and is incident on the nanoantenna arrays, with an area of 4 mm × 105 µm, 7.0 mm × 105 µm, located on the sensing fiber where the poultry sample is placed. The transmitted light is confined in the fiber core and excited due to localized surface plasmon effect leading to the generation of SERS hotspots at the nanogaps between the nano disks, where the interaction between the laser and *Salmonella* samples occurs. The scattered Raman light from the fiber side is reflected back through the coupler, subsequently passing through a notch filter which has similar wavelength, i.e., 532 nm that spectrally filters the light. The filtered light is then collected by the Raman instrument configured for backscattered detection. The signals were acquired using the OceanView software (Orlando, Floria, USA) with a 200 ms integration time for each measurement. The integration time refers to the duration during which the spectrometer collects photons in each scan. This parameter, controlled by the spectrometer’s software, affects the sensitivity and noise level of the measurements. A 200 ms integration time was found to provide better signal quality compared to shorter integration times. This setting remains constant throughout all experiments. The SERS spectrum obtained from the reflection of light from the surface of the SERS sensor allows for the precise resolution and correlation of *Salmonella* or other pathogen samples to Raman wave numbers. For instance, *Salmonella* cells exhibit a vibrational fingerprint/Raman effect attributed to the inelastic scattering of light by the molecular vibrations of *Salmonella* cell components, including proteins, lipids, nucleic acids, and cell wall constituents. This Raman spectrum contains valuable information about the vibrational modes of the molecules which is specific to the sample present in the chamber. The detected *Salmonella* spectrum was then compared with a reference/data base of *Salmonella* and other pathogens spectra, facilitating their identification and detection, i.e., the peaks in the results corresponded to *Salmonella* fingerprint Raman spectra.

### Sensitivity of the biosensor

**Fig. 4:**
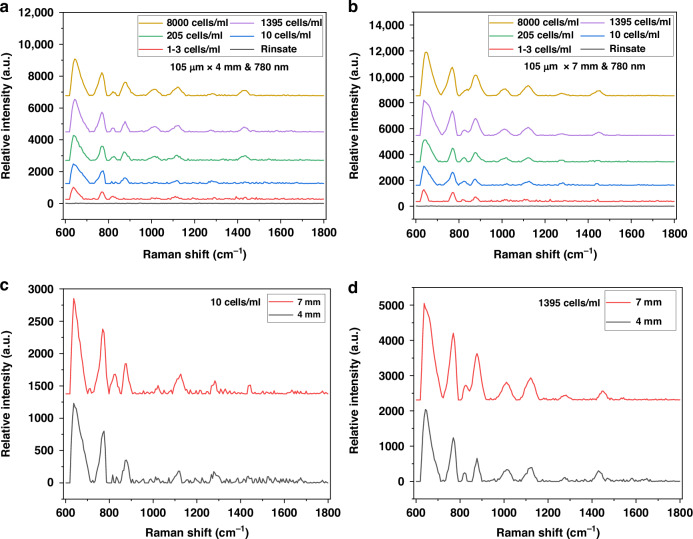
Relative intensity versus concentration for SERS sensor with surface area and disk diameter of (**a**) 105 µm × 4 mm and 780 nm, (**b**) 105 µm × 7 mm and 780 nm, respectively, and versus sensing area for fixed diameter of 780 nm and concentration of (**c**) 10 cells/ml, (**d**) 1395 cells/ml

The optimal sensitivity of the SERS sensor was determined by conducting experiments with *Salmonella* in raw poultry rinsate samples at various concentrations ranging from 1 to 3 cells/ml to 1395 cells/ml, each with a volume of 1 ml. The samples were loaded into the testing chamber, and the SERS spectra were measured before and after loading each sample to obtain the background signal and the total relative intensity signal. The background signal was then subtracted from the total relative intensity signal to obtain the spectra for *Salmonella* alone. Multiple experiments were performed to evaluate the impact of changing the sensing surface area and disk diameter. The sensing surface area was manipulated by adjusting the sensing length, which was set at 4 mm and 7 mm, while maintaining a fixed fiber core diameter of 105 µm. The disk diameters used were 619 nm, 693 nm, and 770 nm. The testing results showed that the relative light intensity increased as the concentration of *Salmonella* increased for both sensing areas of 105 µm × 4 mm and 105 µm × 7 mm, as illustrated in Fig. 4a, b. The figure also includes spectra for chicken rinsate before spiking with *Salmonella*. The spectra do not have any peaks demonstrating that the sample was negative. Moreover, Fig. 4c, d demonstrated that increasing the length of the sensing area, while keeping the disk diameter constant, did increase the interaction between the laser light, *Salmonella*, and the nanoantennas, resulting in a slight rise in relative light intensity. However, this increase was modest, typically under a factor of 2, with only one peak showing an enhancement of around a factor of 3 (Fig. 4). This increase is modest and does not represent an order of magnitude change. A likely explanation is that the 4 mm area was already sufficient for the Salmonella cells to fully spread across the sensing surface. The number of cells detected likely varied slightly, between 1 and 3 cells, which could explain the small fluctuations in signal strength. Expanding the sensing area further is unlikely to provide a meaningful boost, especially given the low cell concentration. Therefore, we conclude that increasing the sensing area beyond 7 mm is unnecessary, as it would not lead to a significant improvement in the SERS signal. Since our goal is to detect very low numbers of cells, a larger area would not provide additional value in this case. Based on this result, the 4 mm length has been selected because it provides enough surface for effective interaction between the laser and bacterial cells. A larger sensing length, such as 7 mm, showed only slight signal improvement and did not lead to an order of magnitude change. This is likely because the 105 μm × 4 mm area already allows sufficient spreading of *Salmonella* cells, especially at low concentrations below 10 cells/ml. This indicates that increasing the sensing area further is unlikely to result in substantial improvements. However, additional investigation is needed to determine the optimal sensing length.

**Fig. 5:**
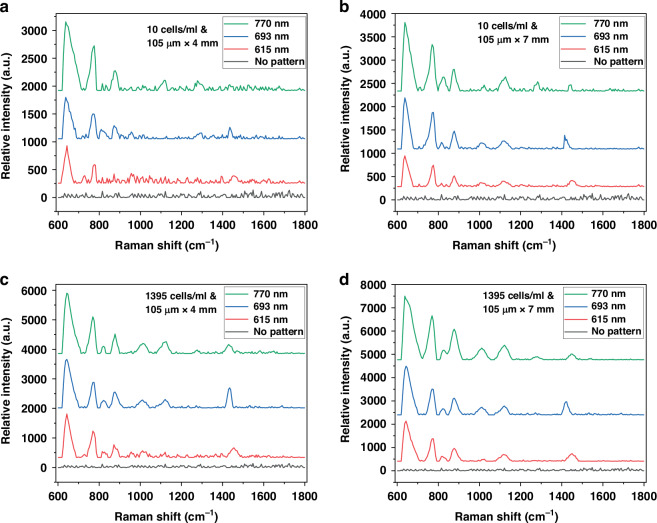
Relative intensity as a function of disk diameters for a fixed concentration of 10 cells/ml for (**a**) 105 µm × 4 mm (**b**) 105 µm × 7 mm, and 1395 cells/ml for (**c**) 105 µm × 4 mm and (**d**) 105 µm × 7 mm

The impact of disk diameters (615 nm, 690 nm, and 780 nm) at fixed periodicity of 1 μm and thickness of 60 nm on sensor performance was investigated, where a larger disk diameter corresponds to a smaller nanogap between the disks. The testing results, as shown in Fig. 5, demonstrate that a larger disk diameter (resulting in a smaller nanogap) for a fixed periodicity leads to a higher relative light intensity SERS signal for the sensors. Figure 5b also includes a spectrum for the sensor without patterning the disk array or depositing gold film. The spectra do not have any peaks demonstrating that Raman signal is very weak. The results were plotted for two sensing areas (105 µm × 4 mm and 105 µm × 7 mm) and two cell sizes (10 cells/ml and 1395 cells/ml). The highest signal was achieved for sensors with a larger disk diameter at a fixed periodicity and larger sensing area, as shown in Fig. 5b, d. The reduction of the spacing between the disks (creating a smaller nanogap) enhanced the electric field, increasing field intensity and forming SERS hotspots via laser excitation between the disk arrays. This resulted in stronger Raman signals from *Salmonella* cells located near these hotspots, thereby improving sensor sensitivity. To achieve stronger SERS signal enhancement, further reduction of the nanogap between the disks to less than 100 nm is required. This could be accomplished by reducing the periodicity by using microspheres with diameters ranging from 0.5 to 0.7 µm. A narrower nanogap will more effectively concentrate the electric field, generating SERS hotspots. As a result, the SERS signals from *Salmonella* or other pathogens near these hotspots will be significantly amplified. The results suggest that several factors—such as sensing surface area, disk diameter, and periodicity—are critical to optimizing the sensitivity of the SERS sensor, through both theoretical modeling and experimental validation.

**Fig. 6:**
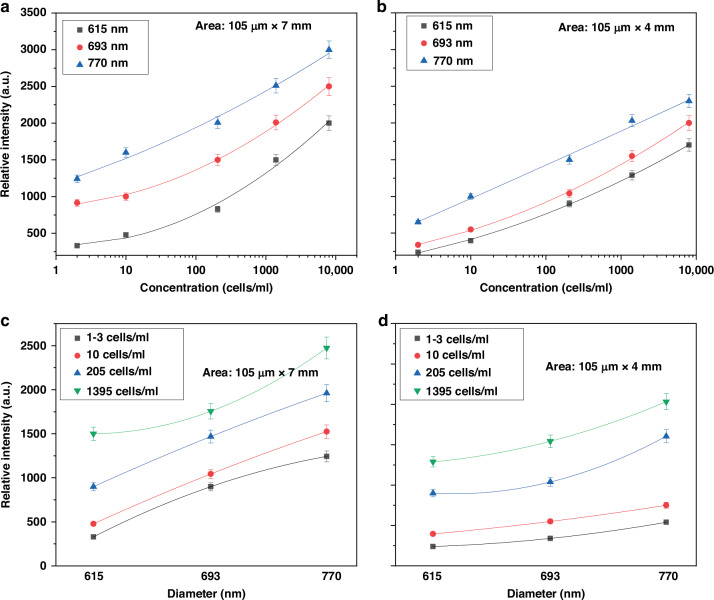
Relative intensity for specific peaks (634 cm^-1^) versus (**a**, **b**) Salmonella concentrations, (**c**, **d**) disk diameters for sensing areas of 105 μm × 7 mm and 105 μm × 4 mm, respectively

To further analyze the results, the peak intensity was plotted as a function of concentration between 1 and 3 cells/ml and 1395 cells/ml, as shown in Fig. 6a, b. The figure demonstrates that the intensity signal increases with an increase in concentration of all three disk diameters. Furthermore, the results were plotted as a function of disk diameter (Fig. 6c, d), revealing that the intensity signal increases with larger disk diameters. The observed trend was logarithmic, which can be explained by the behavior of the cells as their number increases. At lower concentrations, the sensor detects cells efficiently, resulting in a rapid signal increase. However, as the concentration rises, cells may begin to stack or cluster, reducing the effective surface area available for detection. This stacking likely limits further signal increases, even with significantly higher cell concentrations. Moreover, comparing figures with different areas, we observed that the 105 µm × 7 mm sensors exhibit higher sensitivity, confirming our hypothesis.

**Fig. 7:**
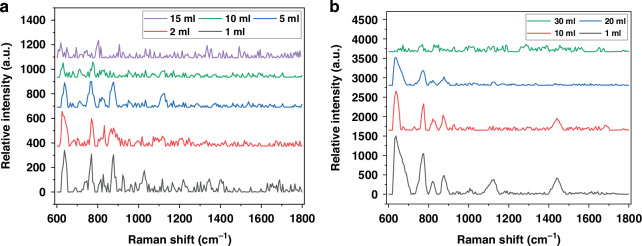
Raman spectra were obtained for *Salmonella* in raw poultry rinsate samples, where the relative Intensity versus volume for (**a**) 1–3 cells, the fiber has a disk diameter and sensing area of 615 nm and 105 µm × 7 mm. **b** 10 cells. The fiber has a disk diameter and sensing area of 770 nm and 105 µm × 7 mm

The *Salmonella* in raw poultry rinsate samples with initial concentrations of 1–3 cells/ml and 10 cells/ml underwent further dilution with carcass rinsate, resulting in final volumes up to 30 ml. Upon testing, the sensor successfully detected *Salmonella* concentrations of 1–3 cells in 5 ml and 10 cells in 20 ml of the prepared diluted samples, respectively. However, the sensor was not able to detect any spectra at a 30 ml dilution, confirming the LoD to be 1–3 cells per 5 ml and 10 cells per 20 ml. At a volume of 20 ml, several peaks disappeared, and increasing the volume to 30 ml caused all peaks to be lost. Signals were recorded 10 min after loading each sample. We observed a slight reduction in signal as the volume increased from 10 ml to 20 ml. At 30 ml, the signal completely disappeared, suggesting that the bacteria were too dispersed within the solution to be detectable by the sensor. These results are plotted in Fig. 7. These findings suggest a sensitivity range of 0.4–0.5 cells/ml.

**Fig. 8:**
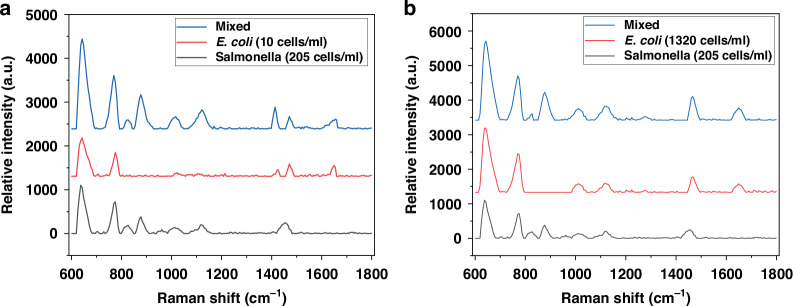
Raman spectra were obtained for mixture of *E. coli* O157:H7 and *Salmonella* in raw poultry rinsate samples with a concentration of (**a**) 15 cells/ml and 205 cells/ml, and (**b**) 1800 cells/ml and 1395 cells/ml, respectively. The figure also plots the spectra for *E. coli* O157:H7 and *Salmonella*, prior of mixing with a concentration of 10 cells/ml and 205 cells/ml, and 1320 cells/ml and 1395 cells/ml, respectively. The tested device has an area and disk diameter of 105 µm × 7 mm and 615 nm

Multiplex detection of multiple pathogens was demonstrated by testing a mixture of *E. coli* O157:H7 and *Salmonella* in raw poultry rinsate samples, as shown in Fig. 8. The concentrations used for the mixture of *E. coli* O157:H7 and *Salmonella* were 15 cells/ml and 205 cells/ml, and 1800 cells/ml and 1395 cells/ml, respectively. The results were plotted and compared with *E. coli* O157:H7 and *Salmonella* spectra prior to mixing, with concentrations of 10 cells/ml and 205 cells/ml, and 1320 cells/ml and 1395 cells/ml, respectively. The comparison, peak by peak, demonstrates that several peaks are specific for *E. coli* O157:H7 (1603 cm^-1^*,* 1411 cm^-1^), others are specific for *Salmonella* (834 cm^-1^*,* 922 cm^-1^*,* 1012 cm^-1^*,* 1131 cm^-1^), and a third set of peaks are not specific for either but are present in both *E. coli* O157:H7 and *Salmonella* spectra (643 cm^-1^*,* 789 cm^-1^*,* 1402 cm^-1^). Therefore, this technique can detect both pathogens simultaneously with high specificity. The results are plotted in Fig. 8.

### Optimum detection time

**Fig. 9:**
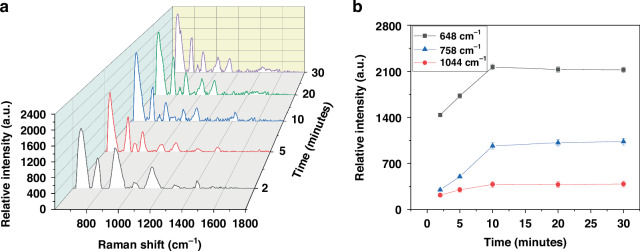
An optimum detection time (**a**) 3D spectrum of the relative Intensity as a function of detection time and wave number, (**b**) relative intensity of specific peaks versus detection time. in this experiment, the fiber core diameter is 770 nm, and the sensing area is 105 µm × 7mm

To determine the optimal detection time, SERS sensor with sensing area and nanodisk diameter of 105 µm × 7 mm and 780 nm, respectively were tested, as shown in Fig. 9. The testing chamber was filled with one ml of *Salmonella* from raw poultry rinsate samples with a concentration of 205 cells/ml and the SERS spectra was measured at different time intervals of 2, 5, 10, 20, and 30 min, as shown in Fig. 9a. Subsequently, we plotted these spectra versus wavenumber and detection time, allowing to observe any changes over time. Additionally, we examined the intensities of several spectral peaks, tracking their variations with respect to time, as shown in Fig. 9b. The figure revealed that the relative intensity of the peaks consistently increased with time until reaching the 10-minute mark. Beyond this point, the signal stabilized, exhibiting no significant increase or decrease in relative intensity. Therefore, we determined that the optimal detection time for *Salmonella* is 10 min.

The experimental results indicated that extending the detection time did not lead to improved sensitivity. Various time intervals were tested, extending up to one hour, but saw no enhancement in the limit of detection. Consequently, the efforts were shifted toward optimizing the sensor’s design to improve its performance. It became evident that the sensor itself plays a more critical role in determining sensitivity. Figure 9 demonstrates that the relative intensity of the signal increased steadily up to the 10-min mark, after which the signal stabilized. Beyond this point, there was no further significant change in intensity. Thus, the 10 min is the optimal detection time for Salmonella. Moreover, the longer detection time did not reduce noise in the spectra. It is noted that the detection time refers to the total elapsed time after placing the sample in the testing chamber and measuring the SERS spectra. The increase in signal over time, up to 10 min, is attributed to the gradual settling of bacterial cells at the bottom of the chamber, concentrating most of them on the sensing area. The bacterial cells along with other debris tend to settle near the bottom of the chamber over time, leading most of them to accumulate at the base of the testing chamber on the sensing area, which is a common phenomenon known in microbiology. About ten minutes after the sample is loaded, the majority of the cells settle at the bottom, stabilizing the signal. However, some cells remain suspended at different levels within the solution, which explains why the sensitivity was measured at around 0.4–0.5 cells/ml.

## Discussion

We have designed, fabricated, and validated a transformative fiber optics-based Surface Enhanced Raman Scattering (SERS) sensor for the rapid detection of Salmonella in raw poultry. To our knowledge, there have been no reports of SERS sensors with nanoantenna arrays fabricated on a side-polished multimode optical fiber core. The sensor design allowed us to insert and fix the fiber into a specially designed 3D-printed plastic microstructure with input and output ports on the sides, and a groove on its top surface, facilitating the polishing of the fiber core and enabling the creation of nanoantenna arrays on the polished surface through the Microsphere Photolithography (MPL) process. The MPL process overcomes the high fabrication costs that have previously impeded the implementation of plasmonic and SERS fiber sensors. The device demonstrated high sensitivity, specificity, and multiplex detection of *Salmonella* and *E. coli O157:H7* in spiked chicken rinsate samples, and detection time.

In this manuscript, increasing the SERS sensing surface area from 105 μm × 4 mm to 105 μm × 7 mm, while maintaining a fixed disk diameter, allowed for more Salmonella cells to land on it, which enhanced the relative light intensity signal, confirming our hypothesis However, in cases of lower Salmonella concentrations, increasing the sensing length did not yield significant signal improvement due to the limited number of cells within the sensing region. However, it is premature to conclude a linear relationship between the SERS signal and the surface area based on just two data points. The results show that the increase in signal is not significant. The testing with two different sensing areas demonstrated the potential impact of area variation on the SERS signal.

Furthermore, employing a larger disk diameter (which resulted in a smaller nanogap) for a fixed periodicity led to a higher relative light intensity SERS signal for the sensors. This is attributed to the smaller nanogap generating a stronger E-field, thus increasing field intensity and forming SERS hotspots through laser excitation between the disk arrays. Consequently, Raman signals from Salmonella cells in the vicinity of these hotspots are enhanced. It is anticipated that further reduction of the gap between the disks, achieved by reducing the periodicity, may result in even higher signal enhancement. These results suggest that the optimal sensitivity of the SERS sensor depends on various factors such as sensing surface area, disk diameter, and Salmonella concentration. Further analysis of peak intensity as a function of concentration, while maintaining fixed diameter and fiber length (sensing surface area), demonstrated a logarithmic increase in signal with concentration. Additionally, the signal also exhibited a logarithmic increase as a function of disk diameter for a fixed concentration of Salmonella cells. This trend can be explained by the behavior of the cells as their number increases. At lower concentrations, the sensor detects cells efficiently, resulting in a rapid signal increase. However, as the concentration rises, cells may begin to stack or cluster, reducing the effective surface area available for detection. This stacking likely limits further signal increases, even with significantly higher cell concentrations.

The sensor’s highest sensitivity, ranging from 0.4 to 0.5 cells/ml, demonstrates its capability to test rinsate samples with volumes much larger than 1 ml. Testing such large volume samples is essential for the poultry industry to achieving rapid testing without the necessity for enrichment culture, all while meeting the Association of Official Analytical Chemists (AOAC) testing requirements for *Salmonella*. This signifies significant advancements in food safety monitoring and has the potential to enhance the detection and prevention of *Salmonella* contamination in poultry products. Furthermore, SERS sensing is specific and suitable for the multiplex detection of foodborne pathogens such as *Salmonella* and *E. coli O157:H7*. This is because the sensor can identify the pathogen type based on its molecular compositions, including proteins, lipids, nucleic acids, and cell wall components of the bacterial cell. For instance, *E. coli O157:H7* exhibits unique peaks at wavenumbers of 603 cm^-1^ and 1411 cm^-1^, whereas Salmonella displays peaks at 834 cm^-1^, 922 cm^-1^, 1012 cm^-1^, and 1131 cm^-1^, with common peaks appearing at 643 cm^-1^*,* 789 cm^-1^*, and* 1402 cm^-1^.

The optimum detection time was 10 min; however, additional testing time did not improve the signal intensity, while less time decreased it. This diagnosis time is significantly lower than the current PCR testing time, which requires 24 h, promising to revolutionize the food safety industry by reducing time to results and enhancing the agility of the poultry sector in making informed decisions regarding intervention actions to improve food safety. Rapid detection could also improve the operation efficiency of the process plans, allowing them to meet United States Department of Agriculture (USDA) performance standards much faster. It enables adjustment of the processing schedule for highly positive flocks to a later time in the day and facilitate more interventions, such as adding more acid or increasing sanitation measures. Additionally, the testing data can be used to monitor trends and identify potential areas for improvement in the process line. This will result in significant cost reductions in sample diagnosis, as products would not need to be stored until negative results are obtained. Processing plants could also strategically test multiple locations across their facilities and implement additional sanitation measures if results are more positive than usual. This would simultaneously improve food safety and expedite intervention actions.

The new SERS sensor will open the door for detection of *Salmonella* serovars and can be readily adapted for the detection of other bacterial and viral pathogens such as *E. coli O157:H7*, *Campylobacter*, *Listeria*, and avian influenza and in other food products, e.g., dairy, beef, and produce, as well as clinical applications.

## Conclusion

This study introduces the design, fabrication, and validation of a transformative fiber optics-based Surface Enhanced Raman Scattering (SERS) sensor for the rapid detection of *Salmonella* in raw poultry. The sensor integrates highly uniform and repeatable nanoantenna arrays onto a side-polished multimode optical fiber core. Initially, the fiber is inserted into a specially designed 3-dimensional (3D) printed plastic microstructure with input and output ports on the sides, and a groove on its top surface. It is then affixed in place using adhesive, facilitating the polishing of both the fiber’s core and cladding. This integration enables the creation of nanoantenna arrays on the polished surface through cost-effective Microsphere Photolithography (MPL) and lift-off processes. The biosensor’s performance is tested using raw chicken rinsates spiked with either *Salmonella* Typhimurium, *E. coli* O157:H7, or a mixture of both pathogens. The results demonstrate that the SERS signal is slightly improved with a larger sensing area for a fixed diameter. Furthermore, the results show that a larger disk diameter, i.e., a smaller nanogap for a fixed periodicity, leads to a higher SERS signal due to the stronger E-field intensity and the formation of SERS hotspots through laser excitation between the disk arrays. Thus, Salmonella cells in the vicinity of the hotspots exhibit a significant increase in Raman signals. The sensor achieves a Limit of Detection (LOD) of 0.4–0.5 cell/ml. To evaluate multiplex detection and specificity, a mixture of *Salmonella* and *E. coli* O157:H7 is tested and compared peak by peak. The results demonstrate that several peaks are specific to *E coli* O157:H7, while others are specific to *Salmonella*, and a third set of peaks is not specific to either but is present in both *E coli* O157:H7 and *Salmonella* spectra. Thus, this technique has the ability to detect both pathogens with high specificity simultaneously. Future research aims to differentiate between various Salmonella serotypes relevant to the poultry industry and other commonly co-occurring bacteria, both pathogenic and nonpathogenic. Time-based studies indicate an optimal detection time of 10 min, beyond which no significant increase or decrease in SERS signal is observed. These findings promise to revolutionize food safety by reducing time-to-results and enhancing the poultry industry’s agility in implementing interventions for improved food safety. Overall, the study presents a groundbreaking fiber optics-based SERS sensor capable of detecting *Salmonella* and *E. coli* O157:H7 with high specificity and sensitivity, showcasing its potential in pathogen detection and food safety applications.
